# Participation of xCT in melanoma cell proliferation in vitro and tumorigenesis in vivo

**DOI:** 10.1038/s41389-018-0098-7

**Published:** 2018-11-14

**Authors:** Seung-Shick Shin, Byeong-Seon Jeong, Brian A. Wall, Jiadong Li, Naing Lin Shan, Yu Wen, James S. Goydos, Suzie Chen

**Affiliations:** 1 Rutgers Cancer Institute of New Jersey, New Brunswick, NJ 08901 USA; 2 Department of Food Science and Nutrition, Jeju National University, Jeju, 63243 South Korea; 3 Susan Lehman Cullman Laboratory for Cancer Research, Ernest Mario School of Pharmacy, Department of Chemical Biology, Rutgers University, Piscataway, NJ 08854 USA; 4 Princeton Enduring Biotech, Princeton, NJ08852 USA

## Abstract

Our research group demonstrated that riluzole, an inhibitor of glutamatergic signaling reduced melanoma cell proliferation in vitro and tumor progression in vivo. The underlying mechanisms of riluzole are largely unknown. Microarray analyses on two human melanoma cell lines revealed that riluzole stimulates expression of the cystine-glutamate amino acid antiporter, xCT (SLC7A11). Western immunoblot analysis from cultured human melanoma or normal melanocytic cells showed that xCT was significantly overexpressed in most melanomas, but not normal cells. Studies using human tumor biopsy samples demonstrated that overexpression of xCT was correlated with cancer stage and progression. To further investigate if xCT is involved in melanoma cell growth, we derived several stable clones through transfection of exogenous xCT to melanoma cells that originally showed very low expression of xCT. The elevated xCT expression promoted cell proliferation in vitro and inversely, these melanoma clones showed a dose-dependent decrease in cell proliferation in response to riluzole treatment. Xenograft studies showed that these clones formed very aggressive tumors at a higher rate compared to vector controls. Conversely, treatment of xenograft-bearing animals with riluzole down-regulated xCT expression suggesting that xCT is a molecular target of riluzole. Furthermore, protein lysates from tumor biopsies of patients that participated in a riluzole monotherapy phase II clinical trial showed a reduction in xCT levels in post-treatment specimens from patients with stable disease. Taken together, our results show that xCT may be utilized as a marker to monitor patients undergoing riluzole-based chemotherapies.

## Introduction

Melanoma is the deadliest form of skin cancer that is derived from the uncontrolled growth of melanocytes derived from neural crest cells. Although the molecular mechanism of melanomagenesis has been extensively studied and several critical genes have been identified, the precise number of genes that are altered and how these changes bring about cell transformation and tumor formation remain elusive and not clearly understood. Our group was the first to suggest a link between glutamatergic signaling and melanoma pathogenesis, subsequently confirmed by others^1–4^. We demonstrated that aberrant expression of metabotropic glutamate receptor 1 (GRM1) in melanocytes was sufficient to induce cell transformation and metastatic tumor formation in vitro and in vivo^5–8^. Since then, GRM1-conditional transgenic mice and transgenic mice with enhanced GRM5 expression displayed a similar metastatic melanoma phenotype^1,2^. In addition, whole-exome sequencing revealed that an ionotropic glutamate receptor, GRIN2A is mutated in 33% (*n* = 52) of melanoma biopsies and cell lines tested^4^. By the same group, GRM3 was also revealed to be mutated in 16.3% (13 of 80 tumors) of melanomas^3^. Taken together, results from these studies support the hypothesis that glutamatergic signaling is a critical factor in the progression of melanomas.

In normal skin, the majority of glutamate release is found not in melanocytes but basal keratinocytes^9,10^. Nevertheless, the functional role of glutamate in melanocytes is very critical. As an example, inhibition of ionotropic glutamate receptors (iGlus) such as AMPAR or NMDAR leads to significant downregulation of MITF, a master regulator of melanocyte differentiation and proliferation^11^. In addition, morphological changes of melanocytes by inhibiting iGlus suggest the presence of strong regulation in glutamate signaling between keratinocytes and melanocytes^10^.

Once transformed, melanoma cells initiate to release excess glutamate by an unknown autocrine mechanism^12^. Indeed, glutamate release in human malignancies is not an uncommon phenomenon. For example, glioma cells display a more aggressive phenotype by releasing excess glutamate which induces neurotoxicity to surrounding neurons^13–16^. Furthermore, breast and prostate cancer cells also release excess amounts of glutamate which confers a growth advantage^7,17–19^. Similarly, inhibition of glutamate release by riluzole, a drug approved by the US FDA for the treatment of amyotrophic lateral sclerosis (ALS), suppresses proliferation of GRM1-positive human tumor cells in vitro and tumor progression in vivo^7,12,18,20^. Despite intensive studies, the precise mechanism and regulation of glutamate release in melanoma cells or how riluzole mediates its suppressive activity in glutamate release are not well elucidated.

In gliomas and/or other neuronal cancers, inhibition of the cystine-glutamate transporter, xCT, reduces the invasiveness of glioma xenografts, probably due to a decrease in glutamate release to the extracellular space resulting in reduced excitotoxic death of neurons via excess glutamate^14^. xCT is an obligate transporter that exchanges intracellular glutamate for extracellular cystine at 1:1 ratio. Once transported, cystine is reduced into 2 molecules of cysteine, the rate-limiting precursor of glutathione (GSH) synthesis. In normal melanocytes, transport of cystine by xCT is utilized for cell proliferation, glutathione production, and protection of cells from oxidative stress. More interestingly, xCT plays a critical role in pheomelanogenesis as shown in subtle grey mice generated by knocking out of xCT gene, *slc7a11*^21^. We proposed that riluzole inhibits the release of glutamate through the glutamate/cystine transporter, xCT. In the absence of xCT, the glutamate release-inhibitory activity of riluzole is much reduced, which is reflected as a decrease of riluzole-mediated anti-proliferative task in GRM1-expressing tumor cells. In the current communication, we established a set of isogenic cell lines where xCT expression was manipulated through genetic means and the consequences of such maneuvering on cell growth in vitro and in vivo were investigated. From these studies, we were able to demonstrate that there is a positive correlation between melanoma cell growth/tumorigenesis and xCT expression levels.

## Results

### Human melanoma cells and biopsies over-express xCT

**Fig. 1:**
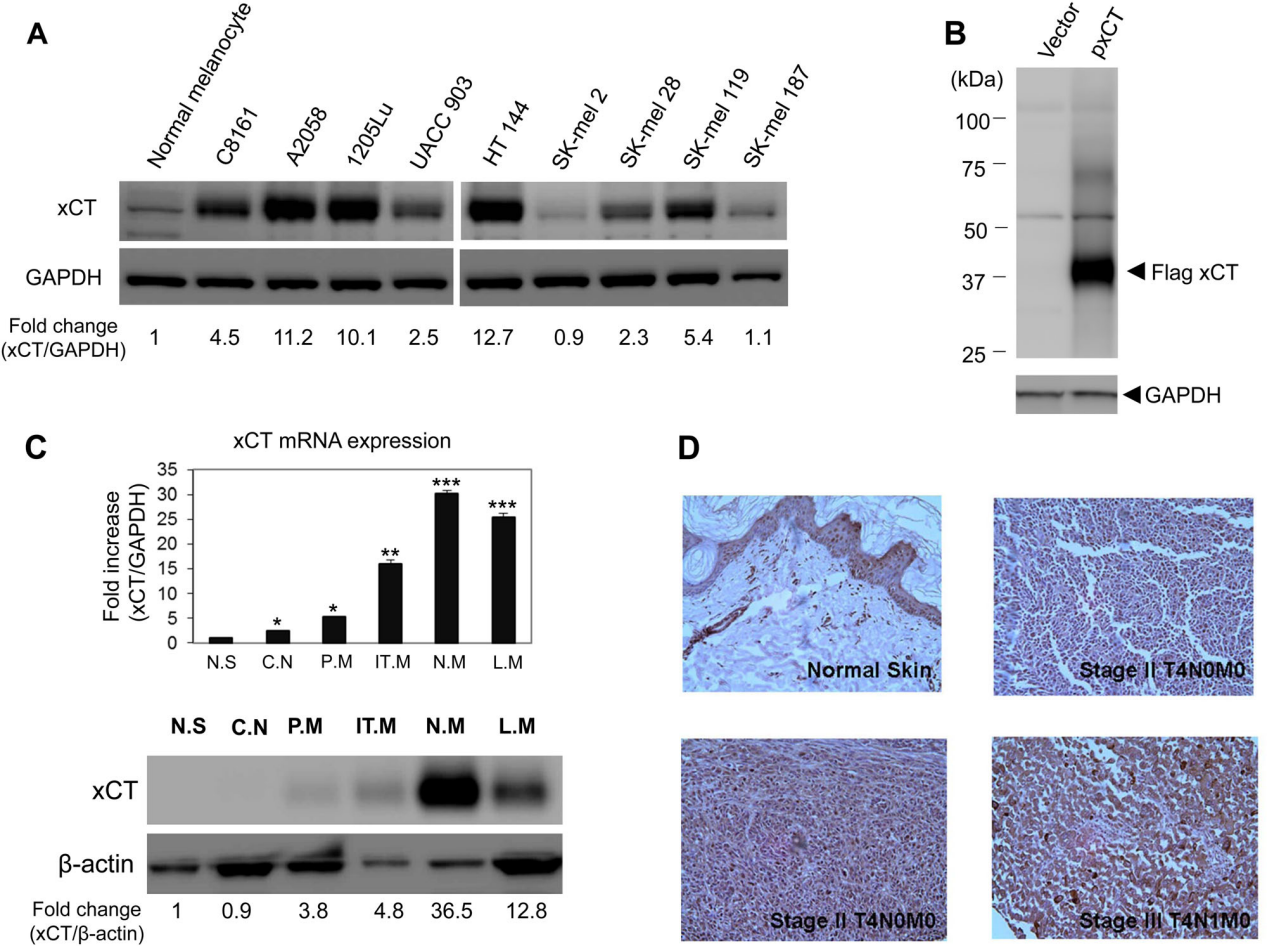
Expression of xCT in human melanoma cell lines and tumors. **a** Western immunoblots were conducted to assess xCT protein expression from one normal human melanocytes and a panel of human melanoma cell lines. Two out of nine melanoma cell lines demonstrated relatively low levels of endogenous xCT protein (SK-mel2 and SK-mel187). Relative expression of xCT was presented as a fold change compared to normal melanocytes. **b** Transient transfection of empty vector and exogenous xCT cDNA in HEK293T cells was performed, followed by western analysis at 48 h post-transfection. GAPDH was used as loading control. **c** An example of xCT expression in human melanoma tumor specimens at various stages. Quantitative RT-PCR was performed to evaluate mRNA expression of xCT in tumor samples. In the bar graph (top panel), the values were presented as the mean ± SEM of three independent experiments. *P < 0.05, **P < 0.01, and ***P < 0.001. Normalization of xCT mRNA to GAPDH levels showed an increase of xCT as the disease progressed. N.S., normal skin, C.N., congenital nevi, P.M., primary melanoma, IT.M., in-transit melanoma, N.M., nodal metastatic melanoma, L.M., lymph node metastatic melanoma. Bottom panel: western immunoblots were conducted to assess levels of xCT protein expression. Relative expression of xCT was presented as a fold change compared to normal skin. N.S., normal skin, C.N., congenital nevi, P.M., primary melanoma, IT.M., in-transit melanoma, N.M., nodal metastatic melanoma, L.M., lymph node metastatic melanoma. **d** Immunohistostaining of xCT on normal skin and tumor sections from stages II and III melanoma patients

Microarray analysis was performed for two different human melanoma cell lines (UACC903 and C8161). Excitatory amino acid transporter1 (EAAT1, also known as SLC1A3 (human) or GLAST [rodent]) and cystine/glutamate transporter (SLC7A11 or xCT) showed the highest relative expression levels among glutamate transporters (Supplementary data, Table S1). Western immunoblots confirmed that xCT is highly expressed in most human melanoma cell lines compared to human primary melanocytes (Fig. 1a). Notably, SK-mel2 and SK-mel187 were two cell lines with lower xCT levels among melanoma cell lines tested. The size of xCT protein was approximately 35kD, which was again verified in HEK293T cells transiently transfected with flag-tagged xCT (Fig. 1b). In addition, we investigated the expression of xCT in human melanoma tumor specimens obtained from patients at varying stages of progression. Quantitative PCR analysis showed that xCT expression positively correlated with the progression of melanomas (Fig. 1c, top panel). This observation was confirmed by western immunoblot analysis which showed elevated xCT protein expression in the tumor biopsies compared to normal skin (Fig. 1c, bottom panel). We also performed immunohistochemical (IHC) staining for xCT in normal and melanoma tissues (Fig. 1d), which confirmed our findings at the RNA and protein levels. Interestingly, in normal skin tissues, some basal keratinocytes were shown to be stained by xCT antibody unlike melanocytes, while melanoma tissues showed very strong staining of xCT throughout the tumor specimen.

### Overexpression of xCT in SK-mel2 and SK-mel187 promotes cell growth

**Fig. 2:**
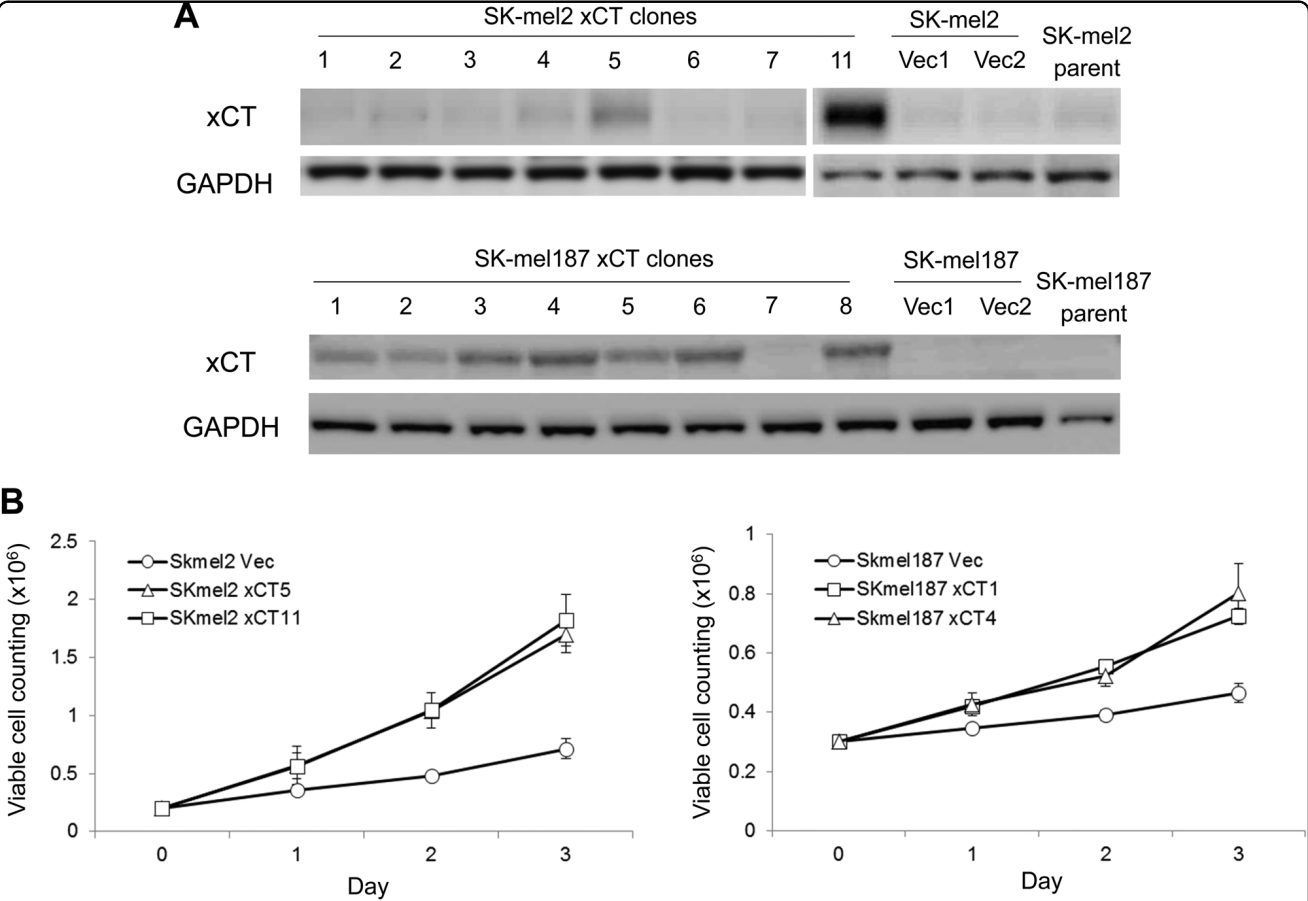
The pro-proliferative effect of xCT in human melanomas. **a** Western immunoblots on protein lysates from several stable clones that have been transfected with either empty vector or exogenous xCT cDNA in two melanoma cell lines (SK-mel2 and SK-mel187) with the lowest endogenous xCT expression (Fig. 1a). **b** Cellular growth was measured on stable xCT or empty vector clones derived from SK-mel2 and SK-mel187 cells. Viable cells were counted using trypan blue exclusion method

Two of the slow-growing human melanoma cell lines SK-mel2 and SK-mel187 showed lower xCT expression levels compared to other melanoma cells (Fig. 1a). We hypothesized that low expression levels of xCT may correlate with the slow-growing property of SK-mel2 and SK-mel187. To test this hypothesis, we introduced either xCT cDNA or empty vector into both cell lines and selected several stable clones from each cell line with varying xCT expression (Fig. 2a). Morphology of these stable clones was similar to the parental cells, except that SK-mel2/xCT clones exhibited a tendency to aggregate and clump together compared to the parental SK-mel2 cells. We selected two xCT clones and one vector clone from each line and examined the growth rate of these clones. These xCT clones from each line (SK-mel2-xCT5, -xCT11, SK-mel187-xCT1, -xCT4) grew much faster compared to clones with empty vectors (SK-mel2-Vec and SK-mel187-Vec) (Fig. 2b).

**Fig. 3:**
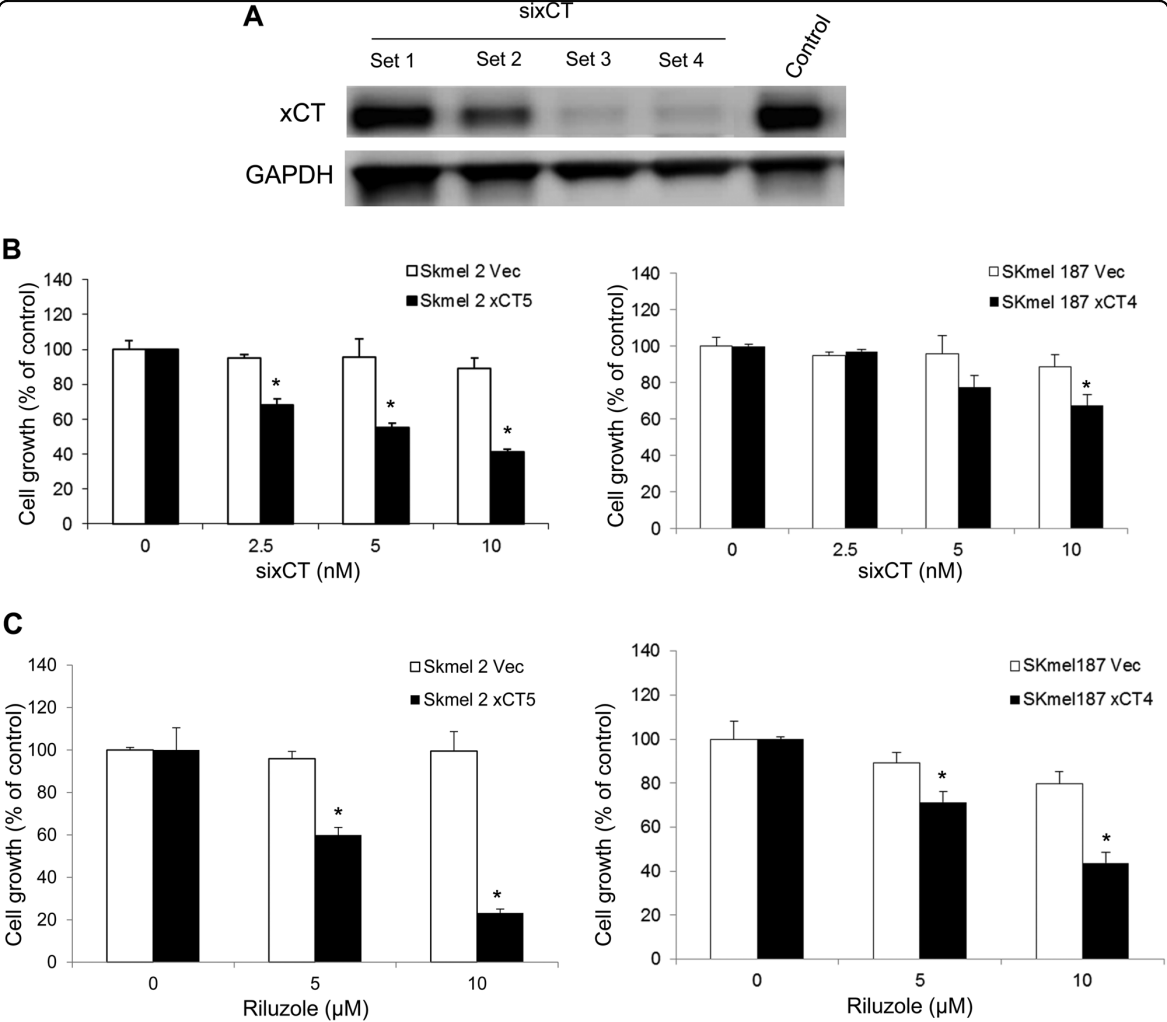
Suppression of xCT expression in xCT stable human melanoma clones by siRNA and riluzole. **a** Knockdown of xCT expression by silencing RNA sixCT. **b** Consequences of reducing xCT expression by sixCT on cell proliferation at day 2 in empty vector controls and exogenous xCT clones derived from SK-mel2 (left panel) and SK-mel187 (right panel). **c** A dose-dependent suppression of cell proliferation in the presence of riluzole in the growth media at (10 μM or 25 μM). For the bar graphs, values are presented as the mean ± SEM from three independent experiments; *P *<* 0.05, compared with the control (no riluzole)

In order to ensure that it was the exogenous xCT promoted cell growth, we selected several commercially available silencing RNA constructs to xCT and assessed their ability to reduce the xCT expression. At 10 nM two of the constructs displayed substantial suppression of xCT expression in the Sk-mel2-xCT11 cell line (Fig. 3a). Set 3 silencing RNA construct (siRNA) was selected for further experiments. The xCT clones were incubated with the siRNA at various concentrations (2.5, 5, or 10 nM) for 48 h, and cell proliferation of SK-mel2-Vec or -xCT5 and SK-mel187-Vec or -xCT4 clones was evaluated by MTS. We demonstrate SK-mel2 xCT5 cells exhibited a significant inhibition of cell growth by silencing RNA constructs in a dose-dependent manner (Fig. 3b, left panel). However, SK-mel2-Vec was not affected by the same treatment (Fig. 3b, left panel). Similarly, SK-mel187-xCT4 displayed reduced growth via the introduction of silencing RNA at 5 or 10 nM concentration; however, the growth of SK-mel187-Vec remained unchanged (Fig. 3b, right panel).

### Enhanced cell survival with elevated xCT expression

We next assessed the sensitivity of the xCT clones to riluzole. One of the functions of riluzole is to reduce the secretion of glutamate from intracellular to the extracellular environment; however, the precise mechanism is unknown. Based on our microarray analysis (Supplementary data, Table S1) we suspected that xCT might be the major player in glutamate transport, where one glutamate is exported and one cystine is imported and reduced to cysteine for participation in glutathione (GSH) synthesis. To test the involvement of xCT in glutamate release, we transiently transfected exogenous xCT into HEK293T cells and conducted glutamate release assays. Our results showed there was approximately 17-fold increase of extracellular glutamate compared to control (Supplementary data, Figure S1), suggesting an association between xCT expression and glutamate release into the extracellular environment. We then performed MTS cell proliferation/viability assays using xCT clones and vector controls derived from each cell line (SK-mel2 and SK-mel187) with riluzole at 5 or 10 μM in the growth media. Notably, these exogenously introduced xCT clones were very sensitive to the treatment of riluzole in a dose-dependent manner; however, vector controls were unchanged (Fig. 3c). From the results of these stable xCT melanoma clones, we concluded that enhanced xCT expression by exogeneous cDNA leads to an advantage in the proliferation of melanoma cells. Conversely, inhibition of xCT using interfering RNA constructs or riluzole significantly limited the proliferation of xCT clones. These results suggest that xCT may be a target of riluzole disturbing the maintenance of the homeostasis with glutamate and cystine levels in melanomas.

### Human melanoma cells with enhanced xCT expression were more tumorigenic in in vivo xenografts

**Fig. 4:**
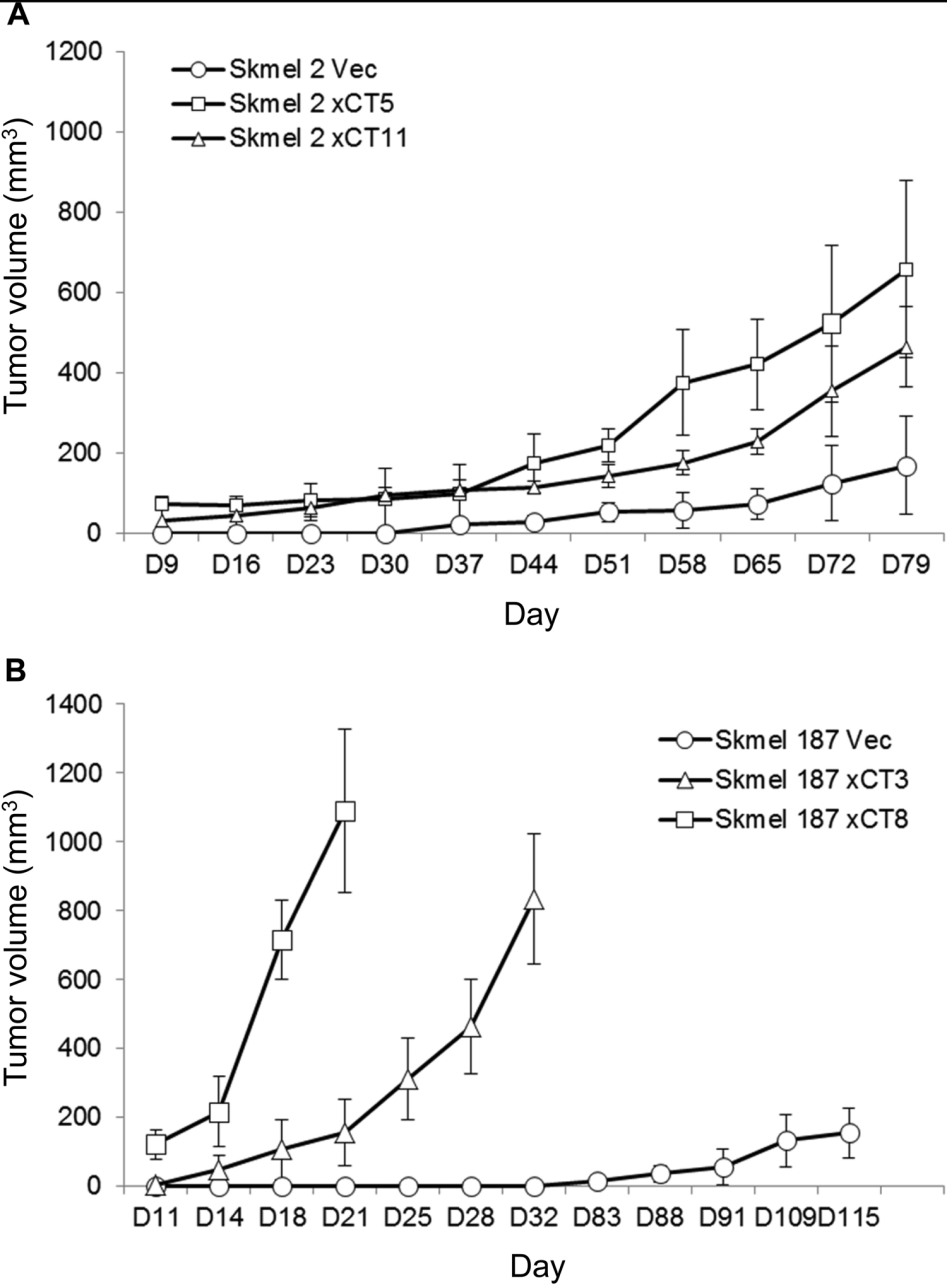
The growth-promoting effect of xCT in human melanoma xenografts. In vivo tumorigenicity assays on vector control and two different xCT clones derived from SK-mel2 (**a**) and SK-mel187 (**b**). One million cells were inoculated into both flanks of 8-week-old nude mice. Tumor volume was measured twice a week

From our observation that enhanced xCT expression is correlated with an increased cell proliferation, we assessed the tumorigenic potential of these xCT cells in in vivo xenograft studies. One vector control and two xCT clones derived from SK-mel2 and SK-mel187 were inoculated into immunodeficient nude mice. In SK-mel2 study, either clone with enhanced xCT (SK-mel2-xCT5 or -xCT11) formed larger xenografted tumors than vector control cells despite the xenografted tumors grew at a relatively slow rate compared to SK-mel187 (Fig. 4a, b). SK-mel187-vector control cells did not form visible tumor until 83 days after inoculation of cells, in contrast, both xCT clones (SK-mel187-xCT-3 or -8) formed robust tumors by day 11, suggesting an association between xCT expression levels and tumorigenesis (Fig. 4b).

### In vivo treatment with riluzole reduced endogenous xCT levels

**Fig. 5:**
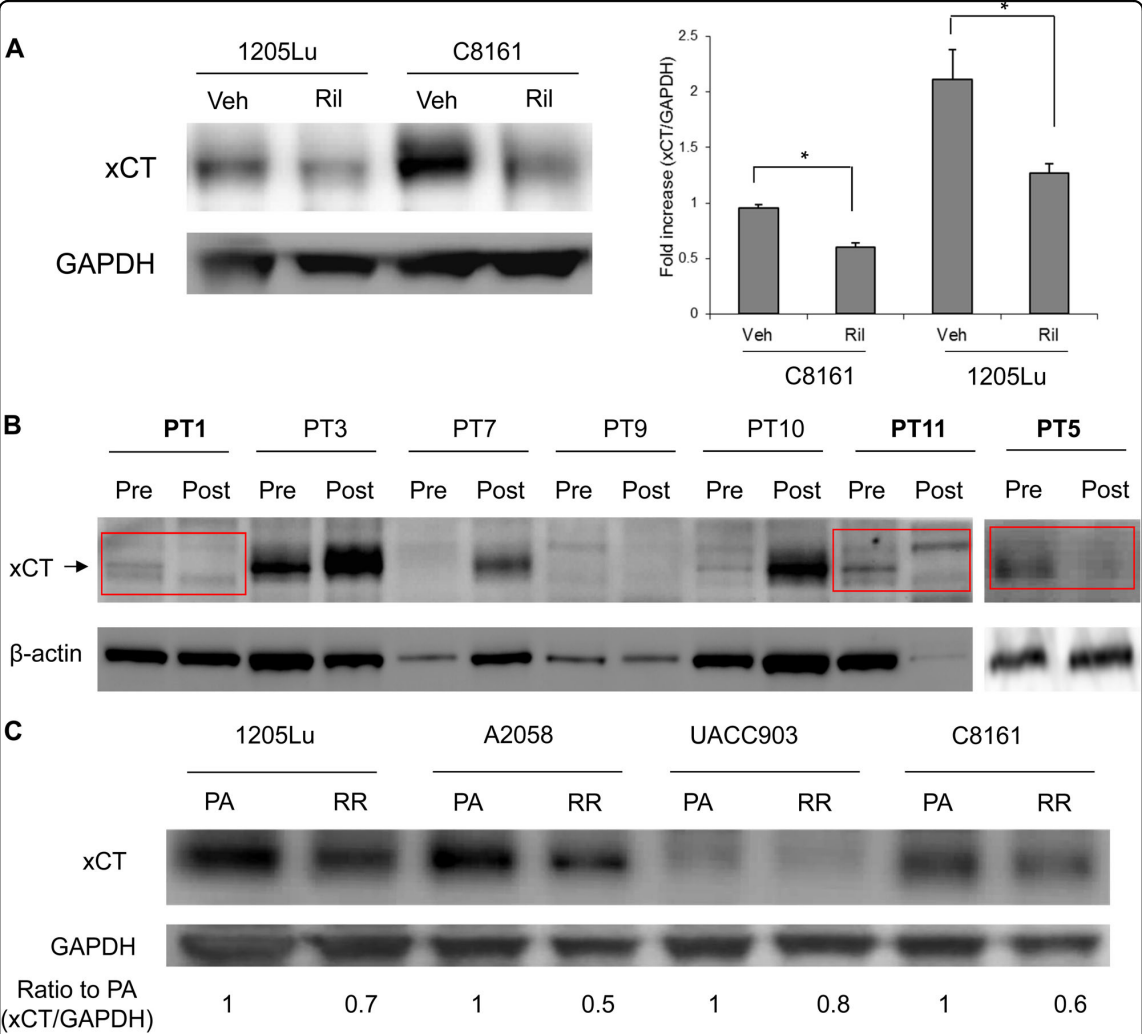
The effect of riluzole on xCT expression in xenograft tumors, riluzole resistant human cell lines, and tumor biopsies from a riluzole monotherapy phase II clinical study. **a** Quantification of endogenous xCT protein expression by western immunoblots using excised xenografts derived from human melanoma cell lines, C8161 and 1205Lu. Xenografted tumors were treated with vehicle (DMSO) or riluzole (7.5 mg/kg) daily by oral gavage for 3 weeks. Western blot data shows decreased level of endogenous xCT protein in riluzole treated samples compared to vehicle-treated ones (left panel). Quantifications were done and normalized to GAPDH (right panel). For the bar graphs, values are presented as the mean ± SEM from three independent experiments; *P *<* 0.05, compared with each control (Veh). **b** Western immunoblots assessing endogenous xCT levels in pre- and post-riluzole treated patient tumors from phase II riluzole monotherapy clinical trial. High endogenous level of xCT in post-treatment tumors are generally correlated with poor prognosis (4/7). Three clinically responded stable disease patients (PT1, PT11, PT5) showed a decreased level of xCT protein in post-treatment compared to that of pre-treatment samples. β-actin was used as loading control. **c** Melanoma cells resistant to riluzole were selected by growing cells in the presence of low concentration of riluzole (5 μM) for 3 passages. Protein lysates were prepared and subjected to western immunoblots. Relative expression of xCT was presented as a fold ratio compared to PA. PA, parent cells, RR, riluzole resistant cells

We performed additional in vivo xenograft studies using two metastatic human melanoma cell lines, C8161 and 1205Lu, that were established previously^12,20,22^. We divided the tumor-bearing mice into two groups: daily treatment with vehicle (DMSO) vs. riluzole (7.5 mg/kg), both for 21 days. As shown previously the riluzole treated group had smaller tumors^23^. Lysates prepared from these tumors showed that endogenous xCT levels were lower in riluzole treated samples compared to vehicle treatment (Fig. 5a).

Based on these preclinical results, we were curious if similar outcomes are observed in patients that participated in a phase II riluzole monotherapy trial^24^. Biopsies from all 13 patients showed increased GRM1 expression independent of B-RAF/N-RAS genotypes confirming our earlier observations in our phase 0 riluzole monotherapy trial^25^. In the initial restaging scan conducted six weeks post-treatment, six of 13 enrolled patients (46%) had stable disease^24^. We performed western immunoblot analysis in seven archived pairs of pre- and post-treatment biopsies and examined endogenous xCT levels. Similar to the result of preclinical xenograft studies (Fig. 5a), a reduction in the endogenous xCT expression levels was detected only in three riluzole-treated patients (PT1, 5, and 11) that achieved stable disease at the 6-week restaging point (Fig. 5b), the same three patients that had reduced pERK and PI3K/AKT activity^24^. To ensure the observation of low xCT levels in biopsies that were responsive to riluzole, we selected riluzole resistant melanoma cells by growing cells under low concentration of riluzole (5 uM) for 3 passages. We examined xCT protein expression in these selected cells compared to the parental cells. As shown in Fig. 5c, cells resistant to riluzole showed lower xCT levels compared to parental cells. These results unequivocally demonstrate that xCT is a target of riluzole.

## Discussion

We had previously demonstrated that aberrant expression of GRM1 (metabotropic glutamate receptor 1) in melanocytes is sufficient to induce spontaneous metastatic melanoma in mice^5^. Recently, there has been increasing activities in the investigation of the glutamatergic system in human malignancies^2,26–29^. Although various molecules including metabotropic or ionotropic glutamate receptors and transporters are involved with glutamate transport via several signaling pathways, it is not known which one is the major player in glutamate release in melanomas. We performed microarray assays using control and riluzole treated samples. From the microarray data, two candidate transporters, xCT, and EAAT1 were identified. EAAT1 is predominantly expressed in the plasma membrane and mediates the transport of glutamate and aspartate into the cells, therefore, we focused on xCT transporter as a candidate implicated in the release of glutamate to the extracellular environment and at the same time uptake of cystine, which is reduced to cysteine and participate in glutathione (GSH) synthesis.

Although most recent research focus on the anti-oxidative action of xCT via GSH production, we hypothesized that glutamate release may be equally important in cancers. Various xCT expression levels were detected in both established melanoma cell lines and tumor tissues. The association between xCT expression and disease progression was not determined in this communication due to the insignificant number of biopsy samples (*n* = 13) examined. Nevertheless, given the results that cell lines such as SK-mel2 and SK-mel187 and biopsy samples, especially primary tumors, displayed very low levels of xCT expression, cellular adaptation on the glutamatergic signaling pathways via xCT may exist.

In the present studies, an association between GRM1 expression and xCT was not observed (Fig. 1a). All melanoma cell lines tested are GRM1-positive but they each have differential xCT expression levels. GRM1 is not detectable in normal human melanocytes while endogenous xCT expression is detectable, suggesting there was no significant difference between GRM1 positive and negative cells with respect to xCT expression. Interestingly, most small molecules inhibitors of xCT such as sulfasalazine and S-4-carboxy-phenylglycine (S-4-CPG) did not alter xCT expression at concentrations of up to 200 μM (data not shown). However, GRM1 agonist, L-quisqualate, was the only compound tested that lowered xCT expression in melanoma cells (data not shown) suggesting that other possible effectors of xCT may be present other than GRM1 such as GRM5 or AMPA receptors.

Riluzole is an FDA approved drug for the treatment of ALS to reduce glutamate release in ALS patients. The precise mechanisms how riluzole mediates the inhibition of glutamate release remain unknown. In this current communication, we demonstrated the close relationship between xCT and glutamate release in transiently transfected HEK cells. Based on these results, we set out to assess the consequences of elevating xCT expression in melanoma cell lines having low endogenous xCT levels.

**Fig. 6:**
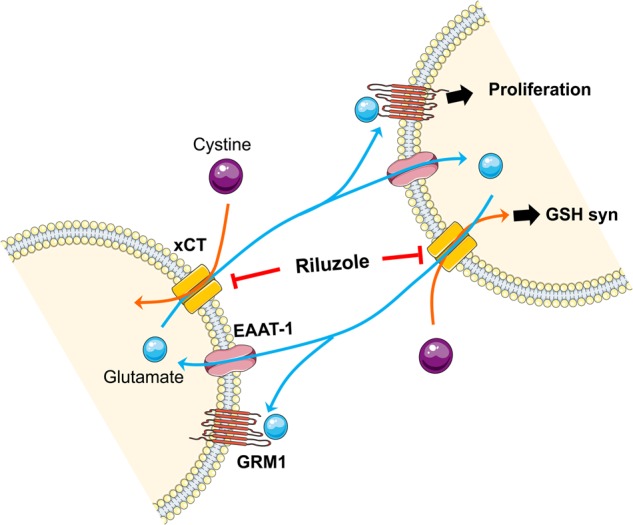
Inhibitory mechanism of riluzole against the glutamatergic system in melanoma. EAAT-1 and xCT participate in the maintenance of glutamate homeostasis in melanoma. Riluzole inhibits glutamate release utilized for both glutamate circulation and GRM1-mediated proliferation in melanoma. xCT, cystine/ glutamate antiporter; EAAT-1, excitatory amino acid transporter 1; GRM1, metabotropic glutamate receptor 1; GSH, glutathione

In normal melanocytes, the balance between differentiation and proliferation seems to be tightly controlled. Meanwhile, in melanomas, glutamate release seems to be utilized for increased proliferation or anti-apoptotic response resulting from mutations in metabotropic glutamate receptors (GRM1, 3, and 5) or ionotropic glutamate receptor (GRIN2A)^2–4,12^. We showed there is an association between xCT levels with cell proliferation in vitro and tumor progression in vivo. The higher the xCT expression level, response of the cells to riluzole is increased, by the addiction of these cells to higher concentrations of glutamate via the xCT system. There has been much interest within the last several years of the contribution of glutamate in the rewiring of cellular metabolisms. Fumagalli and colleagues have reported that riluzole enhanced the glutamate uptake of GLAST (EAAT-1) in a dose-dependent manner in GLAST-transfected HEK cells^30^. In our microarray data using two melanoma cell lines, riluzole enhanced both EAAT-1 and xCT levels. In addition, we demonstrated that in HEK cells transfected with xCT, excess amount of glutamate is released. Taken together, these results suggest that EAAT-1 is involved in glutamate uptake, while xCT participates in the release of glutamate to the extracellular space, proposing coordination of EAAT-1 and xCT in maintaining glutamate homeostasis in melanoma cells. Given that a rapid increase of intracellular glutamate induces cell death in PC12 cells due to an increase of reactive oxygen species (ROS)^31^, riluzole-mediated increase of intracellular glutamate may lead to a similar consequence to melanoma cells. Therefore, in response to a rapid increase of glutamate-mediated oxidative stress, melanoma cells quickly up-regulate xCT expression. Indeed, melanoma cells under oxidative stress due to serum starvation promptly up-regulated xCT protein expression within 2–3 h (data not shown). If some of these melanoma cells survive and acquire resistance to riluzole, they may reduce their dependence on xCT as shown in riluzole resistant melanoma cell lines. Taken together, riluzole may act as an inducer of oxidative stress by inhibiting xCT. Illustration on the proposed mechanism of action of riluzole is shown (Fig. 6).

More recently several reports have linked resistance to B-RAF inhibitors with induced glutamine dependency in melanoma cells^32–35^, suggesting that altered metabolisms and energy sources may be central to acquire drug resistance in some cancers including melanoma. xCT expression in tumor cells has been linked to promotion of cell growth, survival, invasion, metastasis, and resistance to anti-cancer drugs^36–40^. Chan and colleagues performed a set of elegant experiments using genetic manipulation to modulate xCT expression and demonstrated that xCT is a critical component in regulating the nutrient requirement of cells in culture^41^. Levels of xCT expression clearly regulate the preference of the usage of glucose and glutamate particularly during tumor cell growth and limitation on nutrient sources^41^, thus a better understanding on the xCT system in tumor cells may lead to the identification of therapeutic candidate(s) in melanoma.

## Materials and methods

### Chemicals and Reagents

Riluzole (#0768), L-quisqualate (#0188), Sulfasalazine (#4935), S-4-carboxy-phenylglycine (S-4-CPG) (#0322) were purchased from Tocris Bioscience (Minneapolis, MN, USA). G418 (#11811031) was purchased from Gibco (Invitrogen, Carlsbad, CA, USA). Anti-xCT antibody (1:1000, #PA1–16893) antibody was purchased from Pierce Biotechnology (Rockford, IL, USA). Anti-Flag tag antibody was purchased from Origene (1:1000, #TA100011). Anti-GAPDH (1:1000, #5174) and anti-β-actin (1:1000, #4970) antibodies were obtained from Cell Signaling Technology (Danvers, MA, USA).

### Cell cultures

A2058, 1205Lu, HT114, SK-Mel2, and SK-mel28 were purchased from American Type Culture Collection (ATCC) (Manassas, VA, USA). SK-Mel119, SK-Mel187, C8161, and UACC903 were kindly provided by Dr. Suzie Chen. All the cells were cultured in 1x RPMI1640 containing 10% fetal bovine serum (FBS) except for HT144 cell line grown in McCoy’s 5 A containing 10% FBS. Human primary melanocytes were purchased from Coriell Institute for Medical Research (Camden, NJ, USA) and cultured in MelM medium containing melanocyte growth supplement (ScienCell Research Laboratories, Carlsbad, CA, USA) with 5% FBS. 1% Penicillin/Streptomycin was added to the media used. All cell lines were maintained at 37 °C in a humidified 5% CO_2_ incubator. Mycoplasma negativity and cell authentication were tested on a regular basis at the Core Facility (The Biospecimen Repository and Histopathology Service) of Rutgers University.

### Human tumor tissues

De-identified human tumor samples of patients enrolled on a Phase II riluzole monotherapy trial were obtained from Biospecimen Facility Core at Rutgers Cancer Institute of New Jersey by the approval of Rutgers Institutional Review Board (IRB). Informed, signed consent for the use of the tumor samples was obtained from all the patients. Each tumor sample was processed for total protein extraction. Briefly, frozen tissue was grind with mortar and pestle in the presence of liquid nitrogen and the resulting tissue powder was subjected to protein extractions by incubating with Bicine/CHAPS extraction buffer (ProteinSimple, San Jose, CA, USA) on a shaker for 1 h at 4 °C. The samples were then centrifuged at 13,000 × *g* for 10 min and the supernatants were collected. Protein concentration was determined by Piece BCA protein assay kit (Pierce Biotechnology, Rockford, IL USA). 20 μg of total proteins per well were resolved by 4%-12% gradient SDS-PAGE.

### Stable cell line generation

Flag-tagged xCT (SLC7A11) plasmid was purchased from Origene (RC204136) (Rockville, Maryland, USA). xCT plasmid was transfected with Lipofectamin 2000 (InVitrogen. Carlsbad, CA, USA), according to the manufacturer’s instruction. Stably-integrated xCT expressing cells were selected with neomycin and xCT overexpression was confirmed by western blot.

### Immunoblots

Cells were grown to 70–80% confluency, harvested and lysed in Bicine/CHAPS buffer (ProteinSimple, San Jose, CA, USA) in the presence of protease/phosphatase inhibitors. Total protein concentrations were determined by Piece BCA protein assay kit (Pierce Biotechnology, Rockford, IL, USA). A total of 20 μg proteins per well was loaded, transferred onto nitrocellulose membrane, and subsequently probed with xCT and GAPDH antibodies. Specific protein band intensity was quantified using ImageJ software (NIH).

### Quantitative real-time PCR

Total RNA was prepared from either cells or tissues using Trizol reagent (InVitrogen, Carlsbad, CA, USA) and Direct-Zol RNA mini-prep kit (Zymo Research, Irvine, CA, USA), according to the manufacturer’s protocol. Reverse transcription reactions were performed with 1 μg total RNA per 20 μl reaction. Subsequent real-time PCRs were performed in triplicates with Taqman PCR mix (xCT primers #Hs00921933_m1) (Applied Biosystems, Foster City, CA, USA).

### Cell proliferation assay and cell counting

Cell proliferation was measured by CellTiter96 Aqueous Cell Proliferation Assay (Promega, Madison, WI, USA) according to the manufacturer’s instruction. Briefly, cells were seeded in 96-well culture plate at the cell density of 1000 cells/100 μl media/well. Cells were incubated for 1, 2, or 3 days in a humidified 37 °C, 5% CO_2_ atmosphere. MTS solution (10 μl) was added directly to each well and incubated for 2 h and then colorimetric development was measured using Tecan Infinite M200 plate reader (Durham, NC, USA). For viable cell counting, 0.3 × 10^6^ cells were grown on a 35 mm culture dish for 1, 2, or 3 days. Cells were trypsinized and resuspended in 1 ml of media for cell counting using Vi-Cell XR cell viability analyzer (Beckman Coulter, Brea, CA, USA).

### xCT knockdown using siRNAs

The levels of intracellular xCT were knocked down using premade siRNAs of xCT (FlexiTube siRNA, Qiagen, set 1: SI00104895, set 2: SI00104902, set 3: SI00104909, and set 4: SI00104916). Cells at 70–80% confluence were transfected with 0, 2.5, 5, 10, 20 nM of sixCT using Lipofectamine 2000 (InVitrogen, Carlsbad, CA, USA). To ensure the efficacy of sixCT, 10 nM of sixCT was transfected into xCT overexpressing stable clones and cell proliferation was measured at 48 h after the knockdown using CellTiter96 cell proliferation assay kit (Promega, Madison, WI, USA).

### Glutamate quantification

Glutamate concentration in the conditioned media was measured after 48 h in culture with glutamate-free MEM using the Glutamine/Glutamate Determination Kit (GLN1, Sigma-Aldrich, St. Louis, MO, USA) according to the manufacturer’s instructions. The determination of L-glutamate was done by measuring the dehydrogenation of L-glutamate to α-ketoglutarate accompanied by reduction of NAD^+^ to NADH. The conversion of NAD^+^ to NADH was determined by measuring absorbance at 340 nm using a 96-well plate reader (Tecan, Durham, NC, USA). The amount of NADH is proportional to the amount of glutamate in each sample.

### Mouse xenografts

Animal studies were performed by the protocols and criteria approved by the Institutional Animal Care and Use Committee (IACUC) of Rutgers University. Cells for xenograft experiments were grown to 80% confluence, collected, and resuspended in ice-cold 1x PBS. Balb/C female nude mice (4–6-week old) were purchased from Taconic (Hudson, NY, USA). A total of 1 × 10^6^ cells in 100 uL of 1× PBS were injected subcutaneously into each flank of a randomly assigned mouse (*n* = 6 per group). Tumors were measured twice a week for up to 79 days for SK-mel2 and up to 138 days for SK-mel187. None of the mice injected with tumor cells was excluded from the analysis. All the animal studies were performed and analyzed in a randomized manner.

### Immunohistochemistry

The human melanoma tissue array (T386) was purchased from US Biomax, Inc. (Rockville, MD, USA). Immunohistochemical staining for xCT was performed using a rabbit anti-xCT antibody (1:200) (Pierce Biotechnology, Rockford, IL USA) with goat anti-rabbit secondary antibody (1:100, Sigma-Aldrich, St. Louis, MO USA). The IHC procedures were performed by Tissue Analytical Services at Rutgers Cancer Institute of New Jersey.

### Statistical analysis

Before we initiate a given set of experiments we meet with Dr. Lin, a professor in the Biometric Division at Rutgers Cancer Institute of New Jersey to determine the sample size to ensure adequate power for statistical analyses. Values were expressed as average ± S.E.M of three independent experiments. Statistical analysis for two different groups was performed using unpaired Student’s *t*-test. Analysis for more than two groups was performed by one-way analysis of variance followed by Tukey’s multiple comparison test. Statistical analysis was performed using SPSS version 18.0 (IBM, NY, USA). The statistical significance was set at a *P*-value of *P* < 0.05. For in vivo studies, the number of mice (assigned randomly to experiments) was determined as per our consultation with Dr. Lin regarding endpoints (xenografted tumor sizes). We have designed our experiments to detect relatively large differences between experimental groups and control. Consequently, to determine the minimum statistically valid number of mice to use per control group, we used a regression model designed to detect at least a 35% inhibition of tumor growth using an assumption of a standard deviation of 25% of the value of the means for the experimental. For estimating the power/sample size, we assume, based on preliminary results, that the standard deviation of each group will be approximately 25% of the mean of the group (i.e., c.v. = 0.25) at the end of the experiment. With 6 mice per group, the detectable difference is 50% (i.e., ratio of the difference in mean tumor volumes between the tested and the control groups to the mean volume of the control = 35%, with 6 mice per group = 50%).

## Electronic supplementary material

Glutamate release from HEK293T cells transfected with xCT

Relative expression from microarray profiling using two human melanoma cell lines (C8161 and UACC903) treated or not treated with riluzole (25μM)
