# Supplementary figures and images for: Participation of xCT in melanoma cell proliferation in vitro and tumorigenesis in vivo
Source: Oncogenesis. 2018 Nov 14;7(11):86. doi: 10.1038/s41389-018-0098-7 (PMC6234219; doi:10.1038/s41389-018-0098-7)

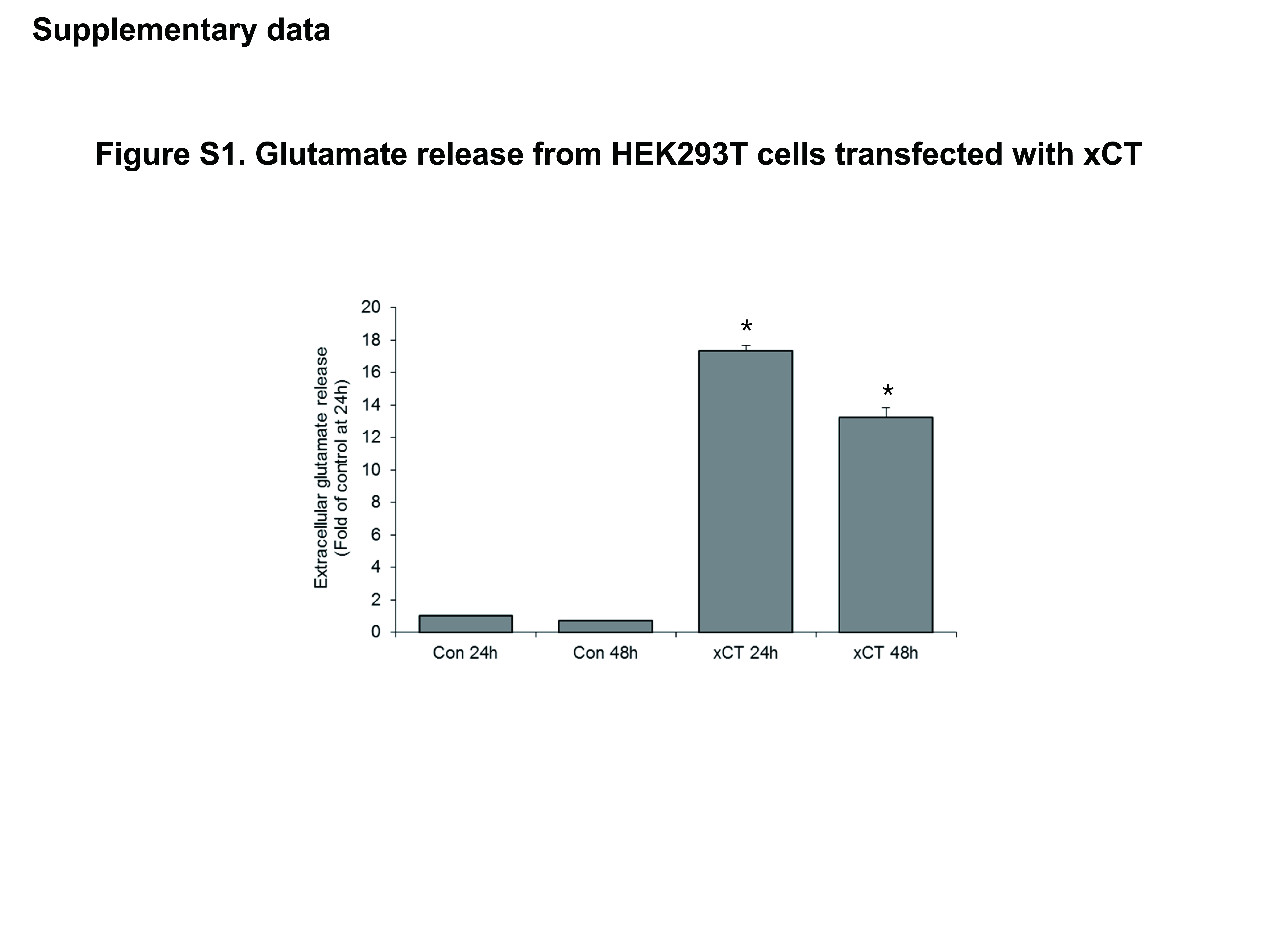

Supplement: Supplementary file 1 — Glutamate release from HEK293T cells transfected with xCT [file 41389_2018_98_MOESM1_ESM.tif]
