# Supplementary material for: Participation of xCT in melanoma cell proliferation in vitro and tumorigenesis in vivo
Source: Oncogenesis. 2018 Nov 14;7(11):86. doi: 10.1038/s41389-018-0098-7 (PMC6234219; doi:10.1038/s41389-018-0098-7)
Supplement: Supplementary file 2 — Relative expression from microarray profiling using two human melanoma cell lines (C8161 and UACC903) treated or not treated with riluzole (25μM) [file 41389_2018_98_MOESM2_ESM.docx]

| **GENE NAME** | **CATEGORY** | **C8161** | **UACC903** |
| --- | --- | --- | --- |
| **GRIN1** | NMDAR | Absence | Absence |
| **GRIN2A** | NMDAR | Absence | Absence |
| **GRIN2B** | NMDAR | Absence | Absence |
| **GRIN2C** | NMDAR | Absence | Absence |
| **GRIN2D** | NMDAR | Absence | Absence |
| **GRIA1** | AMPAR | Absence | Absence |
| **GRIA2** | AMPAR | Absence | Absence |
| **GRIA3** | AMPAR | Absence | Absence |
| **GRIA4** | AMPAR | Absence | Absence |
|  |  |  |  |
| **GRIK1** | KAR | Absence | Absence |
| **GRIK2** | KAR | Absence | Absence |
| **GRIK3** | KAR | Absence | Absence |
| **GRIK4** | KAR | Absence | Absence |
| **GRIK5** | KAR | Absence | Absence |
|  |  |  |  |
| **SLC1A3 (EAAT1)** | Glu Transporters | 208±26 | 74.9±1.1 |
| **SLC1A2 (EAAT2)** | Glu Transporters | Absence | Absence |
| **SLC1A1 (EAAT3)** | Glu Transporters | Absence | Absence |
| **SLC1A6 (EAAT4)** | Glu Transporters | Absence | Absence |
| **SLC1A7(EAAT5)** | Glu Transporters | Absence | Absence |
| **SLC17A7 (VGLUT1)** | Glu Transporters | N/A | N/A |
| **SLC17A6 (VGLUT2)** | Glu Transporters | N/A | N/A |
| **SLC17A8 (VGLUT3)** | Glu Transporters | Absence | Absence |
|  |  |  |  |
| **SLC7A11 (xCT)** | Cys/Glu Transporter | 1093±169 | 135.5±8 |
|  |  |  |  |

**Supplementary Table 1**: Relative expression from microarray profiling using two human melanoma cell lines (C8161 and UACC903) treated or not treated with riluzole (25μM).

Summary of microarray profiling using human melanoma cell lines, C8161 and UACC903 treated with vehicle (DMSO) or riluzole (25μM) for 3 days, RNAs were isolated, purified and used in the array for the identification of proteins participate in glutamate transport. Only EAAT1 and xCT showed significant differences between vehicle and riluzole treated samples in either cell line
